# Associations between thyroid-stimulating hormone and hypertension according to thyroid cyst status in the general population: a cross-sectional study

**DOI:** 10.1186/s12199-020-00910-4

**Published:** 2020-11-05

**Authors:** Yuji Shimizu, Yuko Nabeshima-Kimura, Shin-Ya Kawashiri, Yuko Noguchi, Yasuhiro Nagata, Takahiro Maeda, Naomi Hayashida

**Affiliations:** 1 Department of Community Medicine, Nagasaki University Graduate School of Biomedical Sciences, Nagasaki, Japan; 2 Department of Cardiovascular Disease Prevention, Osaka Center for Cancer and Cardiovascular Disease Prevention, Osaka, Japan; 3 Department of Radiation Health Management, Fukushima Medical University, Fukushima, Japan; 4 Center for Comprehensive Community Care Education, Nagasaki University Graduate School of Biomedical Sciences, Nagasaki, Japan; 5 Department of General Medicine, Nagasaki University Graduate School of Biomedical Sciences, Nagasaki, Japan; 6 Division of Promotion of Collaborative Research on Radiation and Environment Health Effects, Atomic Bomb Disease Institute, Nagasaki University, Nagasaki, Japan

**Keywords:** Cross-sectional study, Hypertension, Normal thyroid function, Thyroid cysts, Thyroid-stimulating hormone

## Abstract

**Background:**

The absence of thyroid cysts may indicate latent thyroid damage, as demonstrated in our previous study. However, the association between the absence of thyroid cysts and latent functional damage of the thyroid is unknown. At low thyroid hormone productivity, which may be associated with latent functional damage of the thyroid, the association between thyroid-stimulating hormone (TSH) and hypertension might be enhanced. Therefore, we evaluated the association between TSH level and hypertension stratified by thyroid cyst status.

**Methods:**

We conducted a cross-sectional study of 1724 euthyroid Japanese individuals aged 40–74 years who participated in an annual health checkup in 2014.

**Results:**

In the study population, 564 and 686 participants had thyroid cysts and hypertension, respectively. A significant positive association was observed between TSH and hypertension in subjects without a thyroid cyst but not in subjects with thyroid cysts. There was a significant positive association between hypertension and TSH in subjects without a thyroid cyst (odds ratio [OR] 1.27; 95% confidence intervals [CI] 1.01, 1.61) but not in subjects with thyroid cysts (OR 0.79; CI 0.57, 1.09) in the model fully adjusted for known confounding factors. The correlation between the TSH and free triiodothyronine (fee T3) levels (simple correlation coefficient [*r*] = − 0.13, *p* < 0.01) was stronger in the subjects without thyroid cysts than in those with thyroid cysts (*r* = − 0.03, *p* = 0.525).

**Conclusions:**

TSH is positively associated with hypertension only in individuals without thyroid cysts. The correlation between the TSH and free T3 levels was stronger in the subjects without thyroid cysts than in those with thyroid cysts. Therefore, the absence of thyroid cysts could be related to the association between TSH level and hypertension, possibly by indicating that the subjects without thyroid cysts had limited thyroid hormone reserves. Therefore, the absence of thyroid cysts could indicate the latent functional damage of the thyroid.

## Background

The absence of thyroid cysts is associated with anti-thyroid peroxidase antibody (TPO-Ab), possibly by resulting in latent thyroid damage [1]. Apart from TPO-Ab, factors such as anti-thyroid antibody and anti-thyroglobulin antibody have also been shown to cause latent thyroid dysfunction. However, the association between the absence of thyroid cysts and latent functional damage of the thyroid is unknown. Additionally, the normal range of TPO-Ab is revealed to have a positive correlation with atherosclerosis among the euthyroid population [2], while atherosclerosis has also been known to be positively associated with hypertension [3, 4]. There could be a significant association between the manifestation of thyroid cysts and hypertension, thereby indicating a lesser influence of latent thyroid damage on hypertension. Although the presence of a thyroid cyst is not clinically significant, the fluid in thyroid cysts was reported to have a high concentration of thyroglobulin [5]. Thyroglobulin is involved in the synthesis and transport of thyroid hormones (triiodothyronine (T3) and thyroxine (T4)) [6]. Thus, the presence of thyroid cysts may have beneficial effects on the production of thyroid hormones. The number of thyroid cysts was reported to increase with growth in school-going age, which is an important period of growth [7]. On the other hand, low thyroid function such as congenital hypothyroidism is associated with slow growth [8].

Depending on the need for thyroid hormone, the pituitary gland secretes the thyroid-stimulating hormone (TSH) into the peripheral blood. In conditions such as hypothyroidism and elevated TSH level in the peripheral blood, a low level of thyroid hormone leads to reduced thyroid function. Studies have reported a positive association between TSH and blood pressure [9, 10]. Both hyperthyroidism and hypothyroidism affect the cardiovascular system and cause diseases such as hypertension [11]. Thus, the presence of thyroglobulin-rich thyroid cysts can affect the association between TSH and hypertension. However, there is no study on the effect of thyroid cysts on the association between TSH and hypertension. To evaluate these associations, we conducted a cross-sectional study of 1724 Japanese individuals aged 40–74 years who participated in an annual health check-up in 2014.

## Methods

### Study population

The study population comprised 1883 Japanese aged 40–74 years from the Saza town in the western part of Japan, who underwent an annual medical check-up in 2014 as recommended by the Japanese government.

To avoid the influence of thyroid disease, subjects with a history of thyroid disease (*n* = 60), without thyroid function data such as TSH, free T3, and free T4 (*n* = 17), and without a normal range of T3 (normal range 2.1–4.1 pg/ml) and T4 (normal range 1.0–1.7 ng/dl) were excluded (*n* = 77).

Additionally, subjects without body mass index (BMI) data (*n* = 1), blood pressure data (*n* = 1), and data for smoking status (*n* = 2) and drinking status (*n* = 1) were excluded. The remaining 1724 subjects with a mean age of 60.5 years (standard deviation (SD) 9.1; range 40–74) were enrolled in the study.

This study was approved by the Ethics Committee of Nagasaki University Graduate School of Biomedical Sciences (project registration number 14051404).

### Data collection and laboratory measurements

A trained interviewer obtained the information for clinical characteristics. Bodyweight and height were measured with an automatic body composition analyzer (BF-220; Tanita, Tokyo, Japan) and BMI (kg/m^2^) was calculated. Systolic blood pressure (SBP) and diastolic blood pressure (DBP) were recorded during rest. Hypertension was defined as SBP ≥ 140 mmHg and/or DBP ≥ 90 mmHg and/or taking anti-hypertensive medication.

The fasting blood sample was collected. TSH, free T3, and free T4 were measured by standard procedures at LSI Medience Corporation (Tokyo, Japan). In addition, HbA1c, triglyceride (TG), and high-density lipoprotein-cholesterol (HDLc) were measured by standard procedures at SRL, Inc. (Tokyo, Japan).

The presence of a thyroid cyst was determined by experienced technicians using a LOGIQ Book XP with a 10-MHz transducer (GE Healthcare, Milwaukee, WI, USA). A cyst (maximum diameter ≥ 2.0 mm) in the thyroid without solid component was defined as a thyroid cyst in this study.

### Statistical analysis

Characteristics of the study population by thyroid cysts were expressed as mean ± SD for continuous variables. Categorical variables such as sex, anti-hypertensive medication use, daily-drinker, and current-smoker were expressed as percent value. Since TSH showed a skewed distribution, the characteristics of the study population were expressed as median [the first quartile, the third quartile] followed by logarithmic transformation. The differences in mean values or proportions of characteristics were analyzed in relation to the presence of thyroid cysts. Significant differences were evaluated with analysis of variance (ANOVA) for continuous variables and with the chi-squared test for categorical values.

Simple and partial correlation analysis of TSH with thyroid hormone (free T3 and free T4) adjusted for age and sex were performed in relation to the presence of thyroid cysts.

Logistic regression models were used to calculate the odds ratios (ORs) and 95% confidence intervals (CIs) and determine the association between hypertension and TSH (tertile and logarithmic values) and the association between subtypes of hypertension (systolic hypertension and diastolic hypertension) and TSH (logarithmic values) in all subjects and in subjects without and with thyroid cyst. In addition, we determined the association between sub-hypertension type (systolic hypertension and diastolic hypertension) and thyroid cyst using logistic regression analysis.

Three adjustment models were used. Model 1 was adjusted only for sex and age; model 2 was further adjusted for free T3 (pg/ml); and finally, model 3 was further adjusted for potential confounding factors including cardiovascular risk factors such as BMI (kg/m^2^), smoking status (current, former, and never), drinking status (non, often, daily), HbA1c (%), TG (mg/dl), and HDLc (mg/dl).

Using analysis of covariance (ANCOVA), the status of thyroid cysts with specific TSH-adjusted free T3 values among non-hypertensive participants were also calculated to evaluate the different levels of thyroid hormones needed based on the status of thyroid cysts among non-hypertensive participants.

All statistical analyses were performed with the SAS system for Windows (version 9.4: SAS Inc., Cary, NC, USA). A *p* value < 0.05 was statistically significant.

## Results

### Characteristics of the study population

The characteristics of the study population stratified according to the presence of thyroid cysts are shown in Table 1. Subjects with thyroid cysts included significantly less male and older individuals who had significantly higher SBP and DBP than those without thyroid cyst.

**Table 1:** Characteristics of study population

		Thyroid cyst		*p*
		(−)	(+)	
Number at risk		1160	564	
Gender of men, %		40.7	29.4	< 0.001
Age, years		59.8 ± 9.4	62.0 ± 8.2	< 0.001
TSH, (0.39–4.01) μIU/ml		1.57 [1.10, 2.29]^*1^	1.60 [1.07, 2.33]^*1^	0.850^*2^
free T3, (2.1–4.1) pg/ml		3.2 ± 0.3	3.2 ± 0.3	0.054
free T4, (1.0–1.7) ng/dl		1.3 ± 0.2	1.2 ± 0.1	0.107
BMI, kg/m^2^		22.9 ± 3.4	22.6 ± 3.3	0.141
Current smoker, %		14.7	11.5	0.068
Daily drinker, %		40.9	39.2	0.506
SBP, mmHg		124 ± 17	126 ± 17	0.005
DBP, mmHg		73 ± 11	74 ± 10	0.045
Anti-hypertensive medication, %		28.7	33.0	0.070
HbA1c, %		5.6 ± 0.6	5.6 ± 0.6	0.074
TG, mg/dl		107 ± 79	101 ± 60	0.113
HDLc, mg/dl		60 ± 15	61 ± 15	0.057

*TSH* Thyroid-stimulating hormone, *T3* Triiodothyronine, *T4* Thyroxine, *BMI* Body mass index, *SBP* Systolic blood pressure, *DBP* Diastolic blood pressure.*TG* Triglycerides, *HDLc* HDL-cholesterol. Values are mean ±standard deviation. *1: Values are median [the first quartile, third quartile]. .*2: Logarithmic transformation was used for evaluating p.

### Correlations between TSH and thyroid hormones

Table 2 shows the correlation between TSH and thyroid hormones (free T3 and free T4) in all subjects and subjects stratified according to the presence or absence of thyroid cyst.

**Table 2:** Correlations between thyroid stimulating hormone (TSH) and thyroid hormones

	free triiodothyronine (free T3)		free thyroxine (free T4)	
	r	(p)	r	(p)
**Total**				
Number at risk	1724			
Simple	-0.10	<0.001	-0.15	<0.001
Partial	-0.09	<0.001	-0.15	<0.001
**Thyroid cyst (-)**				
Number at risk	1160			
Simple	-0.13	<0.001	-0.18	<0.001
Partial	-0.12	<0.001	-0.18	<0.001
**Thyroid cyst (+)**				
Number at risk	564			
Simple	-0.03	0.525	-0.09	0.030
Partial	-0.03	0.418	-0.09	0.030

Partial correlation analysis is adjusted for sex and age. Logarithmic transformation was used for TSH.

For all subjects, TSH had a significant inverse correlation with free T3 and free T4. These correlations were slightly stronger in subjects without thyroid cyst but not significant in subjects with thyroid cysts. In subjects with thyroid cysts, there was a weak but significant correlation between TSH and free T4.

### Associations between hypertension and TSH

The ORs and 95% CIs for the association between hypertension and TSH are shown in Table 3. For all subjects, TSH had a significant positive association with hypertension in model 1 and model 2, but the association was not significant in model 3. The positive association between TSH and hypertension was slightly stronger in subjects without thyroid cyst in all three models. There was no significant association between TSH and hypertension in subjects with thyroid cysts.

**Table 3:** Odds ratios (ORs) and 95% confidence intervals (CIs) for hypertension in relation to thyroid stimulating hormone (TSH)

		Thyroid stimulating hormone (TSH)			*p*	TSH (logarithmic values)
		T1 (low)	T2 (medium)	T3 (high)		
**Total**						
Number at risk		571	579	574		
Number of case		200 (35.0)	231 (39.9)	255 (44.4)		
Model 1		1	1.22 (0.95, 1.59)	1.34 (1.03, 1.73)	0.029	1.23 (1.03, 1.47)
Model 2		1	1.23 (0.95, 1.59)	1.35 (1.04, 1.75)	0.024	1.24 (1.04, 1.48)
Model 3		1	1.15 (0.88, 1.51)	1.15 (0.87, 1.51)	0.324	1.10 (0.91, 1.33)
**Thyroid cyst (-)**						
Number at risk		376	395	389		
Number of case		117 (31.1)	143 (36.2)	174 (44.7)		
Model 1		1	1.26 (0.91, 1.75)	1.62 (1.17, 2.24)	0.003	1.42 (1.14, 1.78)
Model 2		1	1.27 (0.92, 1.76)	1.64 (1.19, 2.28)	0.003	1.44 (1.15, 1.81)
Model 3		1	1.18 (0.83, 1.66)	1.37 (0.97, 1.95)	0.075	1.27 (1.01, 1.61)
**Thyroid cyst (+)**						
Number at risk		195	184	185		
Number of case		83 (42.6)	88 (47.8)	81 (43.8)		
Model 1		1	1.19 (0.78, 1.84)	0.94 (0.61, 1.45)	0.794	0.94 (0.70, 1.27)
Model 2		1	1.19 (0.78, 1.84)	0.95 (0.62, 1.45)	0.804	0.95 (0.70, 1.28)
Model 3		1	1.13 (0.71, 1.77)	0.78 (0.49, 1.24)	0.306	0.79 (0.57, 1.09)

Model 1: adjusted for sex and age. Model 2: + free T3. Model 3: + BMI, smoking status, drinking status, TG, HDLc and HbA1c. Tertile values of TSH for men and women are <1.21 μIU/ml and <1.26 μIU/ml for T1 (low), 1.21-1.89 μIU/ml and 1.26-2.08 μIU/ml for T2 (medium), and 1.90 μIU/ml ≤ and 2.09 μIU/ml ≤ for T3 (high). Case: hypertension. TSH (logarithmic values): ORs and 95% CIs for hypertension in relation to logarithmic values of TSH. ORs and 95% CIs are calculated by logistic regression models.

### Effect of association between TSH and presence or absence of thyroid cysts on hypertension

Since the number of subjects with thyroid cysts (*n* = 564) was smaller than that of subjects without thyroid cyst (*n* = 1160), sample size bias may have influenced the thyroid cyst status-specific association between TSH and hypertension. Therefore, we analyzed the effects of the associations between TSH (logarithmic value) and the two thyroid cysts categories (without and with) on hypertension. Significant effects of the associations were observed, and the adjusted *p* value was 0.024, 0.017, and 0.019 for model 1, model 2, and model 3, respectively (not shown in the table).

### Association between subtype of hypertension and TSH in subjects not taking anti-hypertensive medication

We analyzed the association between the subtype of hypertension (systolic hypertension and diastolic hypertension) and TSH in subjects not taking anti-hypertensive medication **(**Table 4). For total subjects and subjects with thyroid cysts, there was no significant association between TSH and systolic or diastolic hypertension.

**Table 4:** Odds ratios (ORs) and 95% confidence intervals (CIs) for hypertension in relation to thyroid stimulating hormone (TSH) among not-taking antihypertensive medication

		Systolic hypertension		Diastolic hypertension	
		TSH (logarithmic values)	*p*	TSH (logarithmic values)	*p*
**Total**					
Number at risk		1205		1205	
Number of case		154 (12.8)		72 (6.0)	
Model 1		1.31 (0.97, 1.77)	0.078	1.30 (0.86, 1.96)	0.214
Model 2		1.36 (1.00, 1.84)	0.047	1.37 (0.90, 2.08)	0.146
Model 3		1.28 (0.94, 1.73)	0.118	1.32 (0.87, 2.00)	0.189
**Thyroid cyst (−)**					
Number at risk		827		827	
Number of case		91 (11.0)		49 (5.9)	
Model 1		1.62 (1.09, 2.40)	0.017	1.49 (0.90, 2.47)	0.118
Model 2		1.73 (1.15, 2.58)	0.008	1.60 (0.96, 2.67)	0.071
Model 3		1.62 (1.08, 2.44)	0.021	1.57 (0.93, 2.65)	0.092
**Thyroid cyst (+)**					
Number at risk		378		378	
Number of case		63 (16.7)		23 (6.1)	
Model 1		0.95 (0.59, 1.53)	0.824	0.96 (0.47, 1.98)	0.918
Model 2		0.94 (0.58, 1.52)	0.812	0.96 (0.47, 1.88)	0.918
Model 3		0.78 (0.47, 1.30)	0.344	0.75 (0.35, 1.62)	0.465

Model 1: adjusted for sex and age. Model 2: + free T3. Model 3: + BMI, smoking status, drinking status, TG, HDLc and HbA1c. Case: hypertension. TSH (logarithmic values): ORs and 95% CIs for hypertension in relation to logarithmic values of TSH. ORs and 95% CIs are calculated by logistic regression models.

For subjects without thyroid cyst, TSH had a significant positive association with systolic hypertension. Moreover, no significant association between TSH and diastolic hypertension was observed, although there was a positive tendency between TSH and diastolic hypertension.

### Association between subtype of hypertension and thyroid cyst in subjects not taking anti-hypertensive medication

As shown in Table 5, the presence of thyroid cyst had a significant positive association with systolic hypertension but not with diastolic hypertension.

**Table 5:** Odds ratios (ORs) and 95% confidence intervals (CIs) for hypertension in relation to thyroid cyst among not-taking antihypertensive medication

		Systolic hypertension			Diastolic hypertension		
		Thyroid cyst		*p*	Thyroid cyst		*p*
		(−)	(+)		(−)	(+)	
**Total**							
Number at risk		827	378		827	378	
Number of case		91 (11.0)	63 (16.7)		49 (5.9)	23 (6.1)	
Model 1		1	1.51 (1.05, 2.18)	0.025	1	1.10 (0.65, 1.86)	0.728
Model 2		1	1.53 (1.06, 2.20)	0.023	1	1.11 (0.65, 1.87)	0.710
2003Model 3		1	1.61 (1.10, 2.33)	0.013	1	1.12 (0.66, 1.90)	0.685

Model 1: adjusted for age and sex. Model 2: + free T3. Model 3: + BMI, smoking status, drinking status, TG, HDLc and HbA1c. Case: hypertension (systolic or diastolic). ORs and 95% CIs are calculated by logistic regression models.

### Different levels of thyroid hormone needed based on the status of thyroid cysts among non-hypertensive participants

To avoid the influence of over- and under-stimulated thyroid function, TSH-adjusted free T3 values in subjects without hypertension were calculated. We found that subjects with thyroid cysts (*n* = 312) had significantly lower values of free T3 (least mean square ± standard error 3.13 ± 0.02 pg/mL) than those in subjects without thyroid cyst (*n* = 726) (3.18 ± 0.01) (*p* = 0.021) (not shown in the table).

## Discussion

This study shows that TSH had a significant positive association with hypertension only in subjects without thyroid cyst. Therefore, the absence of thyroid cysts could be related to the association between TSH level and hypertension, possibly by indicating the latent functional damage of the thyroid.

Previous population-based cross-sectional studies reported a positive association between TSH and blood pressure (both systolic and diastolic) [9]. Another study reported that TSH had a positive association with diastolic blood pressure but not with systolic blood pressure [10]. Our previous study with a euthyroid population revealed a significant positive association between hypertension and subclinical hypothyroidism among participants without thyroid cysts, but not among those with thyroid cysts [12]. In this study, we found further evidence that TSH was positively associated with both systolic hypertension and diastolic hypertension only in subjects without thyroid cyst (Table 4).

**Fig. 1:**
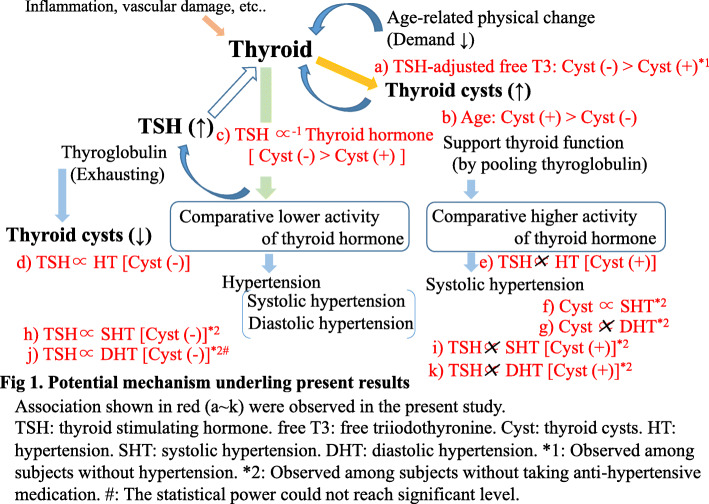
Potential mechanism underling present results

The potential mechanism underlying the present results is shown in Fig. 1. Thyroid cysts might support thyroid function, possibly by pooling thyroglobulin.

Thyroid cysts are reported to be rich in thyroglobulin [5] which plays a crucial role in synthesizing and transporting thyroid hormones [6]. Therefore, subjects with thyroid cysts might have a higher capacity of producing thyroid hormone than those without thyroid cyst. Furthermore, because decreased thyroid function may lead to extended longevity, aging processes might decrease the demand on thyroid hormone activity [13]. Therefore, thyroid cysts might be formed during aging partly due to comparatively excess levels of thyroid hormone. Congruent to these observations, participants with thyroid cysts were significantly older than those without thyroid cyst in the current study (Table 1, Fig. 1 b). However, there was no significant difference in thyroid function as gauged by the levels of free T3, free T4, and TSH (Table 1). The absence of thyroid cysts may indicate latent thyroid damage [1] since TPO-Ab, which is a known cause of autoimmune thyroiditis, is inversely associated with thyroid cysts among the euthyroid population. Moreover, a significant inverse correlation was observed between TSH and free T3 in subjects without thyroid cyst (sex-and age-adjusted partial correlation coefficient (*r*) = − 0.12, *p* < 0.001) but not in subjects with thyroid cyst (*r* = − 0.03, *p* = 0.418) (Table 2, Fig. 1 c). Furthermore, all subjects showed a significant inverse correlation between TSH and free T4, and the magnitude of this correlation was stronger in subjects without thyroid cyst (*r* = − 0.18, *p* < 0.001) than in those with thyroid cysts (*r* = − 0.09, *p* = 0.030) (Table 2, Fig. 1 c). As a result of negative feedback, high levels of T3 and T4 inhibit the TSH secretion from the pituitary gland. The stronger correlation between TSH and thyroid hormone observed in subjects without thyroid cyst than in those with thyroid cysts likely indicates that subjects without thyroid cysts had limited reserves of thyroid hormone. The capacity of producing thyroid hormone in subjects without thyroid cyst is weaker than in subjects with thyroid cysts. Elevated levels of TSH could lead to a more severe reduction in thyroid hormone activity in subjects without thyroid cyst than in those with thyroid cysts. Therefore, the positive association observed in subjects without thyroid cyst (Table 3, Fig. 1 d) can be due to the reduced activity of thyroid hormone. Furthermore, there was no significant association between hypertension and TSH among participants with thyroid cysts (Table 3, Fig. 1 e). These results indicate that the TSH levels of participants with thyroid cysts were no longer associated with the activity of thyroid hormone.

Both hypothyroidism and hyperthyroidism were reported to affect the cardiovascular system and increase the risk of hypertension [11, 14, 15]. An increase in diastolic blood pressure was uncommon in hyperthyroidism because of the reduction in systemic vascular resistance [14, 16]. Hypothyroidism was reported to be associated with increased diastolic blood pressure [11, 15]. Additional analysis in subjects not taking anti-hypertensive medication showed that thyroid cyst had a significant positive association with systolic hypertension but not diastolic hypertension (Table 5, Fig. 1 f, g). Therefore, subjects with thyroid cysts can have a higher activity of thyroid hormone than those without thyroid cyst. Furthermore, this study showed that both systolic hypertension and diastolic hypertension were positively associated with TSH in subjects without thyroid cyst but not in subjects with thyroid cysts (Table 4 Fig. 1 h–k). These results support the above-mentioned mechanisms.

Although thyroid cysts can have a beneficial effect on the production of thyroid hormones, there was no significant difference in the thyroid hormone levels between subjects without and with thyroid cyst in this study population. This paradoxical phenomenon can be explained by the different levels of thyroid hormone needed by different individuals. To avoid the influence of over- and under-stimulated thyroid function, we calculated the TSH-adjusted free T3 values in the subjects without hypertension because these values could indicate the demand value of the thyroid hormone. We found subjects with thyroid cysts (*n* = 312) had significantly lower values of free T3 (least mean square ± standard error 3.13 ± 0.02 pg/ml) than those without thyroid cyst (*n* = 726) (3.18 ± 0.01) (*p* = 0.021) (Fig. 1 a).

This study is clinically significant since the results showed that the thyroid cyst, which is generally regarded to lack clinical significance, can be a determinant factor in the association between TSH and hypertension; higher TSH levels may indicate lower thyroid function that is associated with hypertension only among participants without thyroid cyst. Moreover, the presence of thyroid cysts could indicate high risk of systolic hypertension due to high production of thyroid hormone. Therefore, the absence of thyroid cysts is associated with latent functional damage of the thyroid. In addition, even further investigation is necessary, as these results indicate that the methods for anti-hypertension might be quite different between patients with or without thyroid cysts, even among euthyroid patients; for participants with thyroid cysts, thyroid hormone inhibitors are necessary, whereas for participants without thyroid cyst, supplements of thyroid hormone may be effective.

Unlike regular epidemiological studies, the current work performed a multi-faceted analysis in a general population and the obtained results explained a single mechanism. While subclinical thyroid disease (characterized by normal levels of the thyroid hormone but elevated or decreased TSH levels) is known to be associated with cardiovascular disease [17], the precise mechanisms contributing to the same are yet to be understood. By analyzing the status of thyroid cysts, the present study indicates that a comparatively higher activity of the thyroid hormone is associated with hypertension. The relatively higher activity of the thyroid hormone can be estimated by the strength of the association between TSH and thyroid hormone and not by the levels of thyroid hormone alone. These results could provide efficient cues to clarify the mechanistic details of cardiovascular risks associated with subclinical thyroid disease.

This study has potential limitations. First, we evaluated only the presence or absence of thyroid cyst and did not consider the number and size of cysts. Second, the presence of anti-thyroglobulin antibody causes a marked decrease in thyroglobulin level [5]. Although the anti-thyroglobulin antibody might be a strong confounding factor, we could not measure anti-thyroglobulin antibody because of the limited amount of blood sample. Third, the turnover of thyroid hormone may be different in non-atherosclerotic subjects with thyroid cysts, but we could not evaluate the half-life of thyroid hormone. Fourth, this was a cross-sectional study, and thus, causal relationships could not be established. In future, studies should be conducted after taking these factors into consideration.

## Conclusion

In conclusion, TSH is positively associated with hypertension only in individuals without a thyroid cyst. And the correlation between the TSH and thyroid hormone levels was stronger in the subjects without thyroid cysts than in those with thyroid cysts. Therefore, the absence of thyroid cysts could be related to the association between TSH level and hypertension, possibly by indicating that the subjects without thyroid cysts had limited thyroid hormone reserves. Present results bring a new perspective that thyroid cyst could act as a marker for the activity of thyroid hormone.

## Data Availability

The datasets generated during and/or analyzed during the current study are not publicly available due to ethical consideration but are available from the corresponding author on reasonable request.

## Abbreviations

TSH: Thyroid-stimulating hormone
OR: Odds ratio
CI: Confidence intervals
T3: Triiodothyronine
T4: Thyroxine
TPO-Ab: Anti-thyroid peroxidase antibody
BMI: Body mass index
SD: Standard deviation
SBP: Systolic blood pressure
DBP: Diastolic blood pressure
TG: Triglyceride
HDLc: High-density lipoprotein-cholesterol
ANOVA: Analysis of variance
*r*: Correlation coefficient
ANCOVA: Analysis of covariance
